# Detecting and Locating Passive Video Forgery Based on Low Computational Complexity Third-Order Tensor Representation

**DOI:** 10.3390/jimaging7030047

**Published:** 2021-03-05

**Authors:** Yasmin M. Alsakar, Nagham E. Mekky, Noha A. Hikal

**Affiliations:** Department of Information Technology, Faculty of Computers and Information Science, Mansoura University, Mansoura 35516, Egypt; nagham@mans.edu.eg (N.E.M.); dr_nahikal@mans.edu.eg (N.A.H.)

**Keywords:** inter-frame forgery, digital forensics, correlation, SVD, Harris, GLCM, Tensor, video forensic

## Abstract

Great attention is paid to detecting video forgeries nowadays, especially with the widespread sharing of videos over social media and websites. Many video editing software programs are available and perform well in tampering with video contents or even creating fake videos. Forgery affects video integrity and authenticity and has serious implications. For example, digital videos for security and surveillance purposes are used as evidence in courts. In this paper, a newly developed passive video forgery scheme is introduced and discussed. The developed scheme is based on representing highly correlated video data with a low computational complexity third-order tensor tube-fiber mode. An arbitrary number of core tensors is selected to detect and locate two serious types of forgeries which are: insertion and deletion. These tensor data are orthogonally transformed to achieve more data reductions and to provide good features to trace forgery along the whole video. Experimental results and comparisons show the superiority of the proposed scheme with a precision value of up to 99% in detecting and locating both types of attacks for static as well as dynamic videos, quick-moving foreground items (single or multiple), zooming in and zooming out datasets which are rarely tested by previous works. Moreover, the proposed scheme offers a reduction in time and a linear computational complexity. Based on the used computer’s configurations, an average time of 35 s. is needed to detect and locate 40 forged frames out of 300 frames.

## 1. Introduction

Recently, recording videos using digital cameras, smartphones, and surveillance camcorders has become very easy and has been performed for many reasons in our everyday activities. Millions of videos are available every day, either uploaded over different internet sites or shared among social media. However, any video is easy to create or forge due to the widespread use of software video editing applications. Any editing video software can be used to tamper with videos such as Adobe Video Editor, Photoshop, Premiere by Adobe, and Windows Movie Maker, which are really good methods to easily edit video content, as anyone can edit the video files as it will be similar to the original content. These software applications have made forgery identification very difficult and have led to serious issues. Recently, detecting forged videos has gained great interest and has become a trending research topic compared to video authentication but authenticating the video contents may be unavailable all the time [1,2].

Digital video consists of a large group of sequential images, also known as frames, displayed in rapid succession to create the illusion of motion. Any malicious tampering in video content that alters its visual meaning is considered video forgery. Fast transition between scenes can be easily distinguished from forgery [3]. Video Forgery is categorized into three types regarding its operations domain. The first type is intra-frame forgery, also called a copy-move attack, this happens in the spatial domain, where certain objects are copied and pasted from one region to another within the same frames [4]. The second type is spatiotemporal domain forgery, called a region splicing attack, which occurs when some objects are copied from some frames and pasted onto other frames [5]. The last type is inter-frame, which occurs in the temporal domain if some frames are deleted from the original video (frame deletion), inserted from another video (frame insertion), or duplicated from the same video (frame duplication) [6]. In actuality, the first two types can be easily observed by the human eye, since the movement of forged objects through frames mostly fails to achieve smooth transitions. Inter-frame forgeries have gained researchers’ interest due to their great implications and detecting challenges.

Video forgery detecting methods are categorized into active and passive methods [7]. Active methods are based on analyzing certain types of embedded authentication information inside the original video, such as watermarks or digital signatures. This information is reviewed and checked to prove the correctness of the videos. Fake videos are those that failed in the authentication process. However, most of the videos are not protected by authentication information. Therefore, passive approaches have become necessary as they are more flexible, robust and effective. Passive methods trace video frames searching for signs of forgery, such as: insertion, duplication, deletion, and replacement of frames into original videos. Moreover, passive methods can detect different types of forgeries and localize them.

Throughout the state-of-the-art methods, passive approaches work on video frames one-by-one in the spatial domain to detect signs of forgery. They compare all successive video frame features and depend on spatial correlation measures to prove the discontinuity of frame sequences. These features limit passive approaches performance in terms of detection time and accuracy, especially in the case of large video sizes with a low content variation. Recently, tensor data representation has been considered a trend computational approach to deal with large videos, it provides greater model fitting stability, easier to read and saves time [8].

The offered approach in this paper develops a new inter-frame forgery passive approach that has high efficiency in respect to the achieved detection accuracy at minimum computational complexity. The main idea is as follows:

- The method is based on comparing a limited number of orthogonal-features extracted from third-order tensor video decomposition;
- First, the whole video sequence is geometrically constructed into sub-groups, and each sub-group is mathematically decomposed into a group of third-order tensors. Then, instead of comparing all the frame/feature correlations, a group of arbitrarily chosen core sub-groups is orthogonally transformed to obtain essential features to trace along the tube fibers. Moreover, if a forgery is detected, these features can be used to localize the forged frames with high accuracy;
- The novelty of this paper is the great accuracy in detecting inter-frame forgeries. Hence, the geometric construction of successive video frames into third-order tensor tube fiber mode offers a great reduction in the number of pixels needed to trace forgeries;
- Checking one or two core sub-groups/third-order tensors of a limited number of pixels in the orthogonal domain is enough to detect frame discontinuities, compared with classic passive methods that examine the entire frame sequences. Additionally, this construction encapsulates the spatial and temporal features of successive frames into 2D matrices which can be manipulated and tested easily with high accuracy and less computational complexity.

The following paper structure is outlined as follows: Section 2 discusses the related work on passive video forgery methods. Section 3 introduces a comprehensive analysis of the proposed method. Section 4 presents the experimental investigation results of the proposed method. A comparison and analysis of the results are given in Section 5. Finally, in Section 6, the conclusions and future directions are introduced.

## 2. Related Work

Many important research developments have been made around digital video forensics. In this section, a summary of related research on passive approaches is introduced. Passive approaches trace video frames searching for three types of forgery: multiple/double compression, region tampering, and inter-frame video forgery. This proposed paper mainly considers the inter-frame forgery type in detail.

Inter-frame video forgeries occur by inserting, deleting and duplicating frames in a video. Many studies that worked on inter-frame types faced problems such as accuracy and complexity of detecting and locating. Previous studies worked by comparing successive frames and found that they required a long time for video forgery detecting and locating regardless of forgery type. The most commonly used techniques in the studies were handcrafted methods [9] that depend on different methods of manual extraction of features from video frames. There are many methods for extracting various types of features from video frames. Forgery has been identified according to the stability of the characteristics detected for the specific problem such as frame duplication, frame deletion, frame insertion–deletion and insertion–deletion–duplication. Inter-frame forgery case-related research is introduced in the following sections.

In the case of frame duplication detection, Yang et al. [10] solved frame duplication forgeries using an effective two-stage method. It calculated the similarities using the correlation coefficient between Singular Value Decomposition (SVD) features extracted from each frame. Singh et al. [11] identified duplicated frames from video by extracting nine characteristics for each frame and then lexicographical sorting was carried out to group similar frames. Between these characteristics, Root Mean Square Error (RMSE) was calculated. To recognize the duplicated frames, the correlation between frames was calculated.

For the frame deletion cases, Liu et al. [12] detected frame deletion by analysis of its time and frequency domain features and measuring the periodicity of the Sequence of Average Residual of P-frames (SARP) of videos with frames deleted, SARP results were represented in spikes at certain positions in the Discrete-Time Fourier Transform (DTFT) spectrum. YU et al. [13] detected frame deletion by presenting two features to measure the prediction residual variation magnitude and intramacro block number.

For the case of frame deletion and insertion, Wang et al. [14] depended on computing the consistency of correlation coefficients of gray values (CoGVs) and then fed them into Support Vector Machine (SVM) to classify forged and original videos. Zhang et al. [15] proposed a sequence to detect frame deletion and insertion using two steps, In the first step, the correlation was calculated for Local Binary Patterns (LBPs) of every frame and in the second step, abnormal point detection was applied using the Chebyshev inequality twice. Aghamaleki and Behrad [16] identified frame insertion or deletion, mathematically analyzing the quantization error traces of P-frame residual errors. An algorithm was then proposed to classify rich areas of quantization-error in the P-frame. A wavelet-based algorithm was addressed to enrich the quantization error traces in the frequency domain. These interpreted and spatially limited residual errors are used to detect video forgery in the temporal domain.

For the case of frame deletion, insertion and duplication cases, Bakas et al. [6] detected frame duplication insertion and deletion in videos. They extracted outlier frames using correlation and then used finer levels to eliminate false positives from the first level. Zhao et al. [17] focused on similarity analysis and passive blind forensics scheme for shots of videos was analyzed to identify inter-frame type forgeries. This method consisted of two parts: Hue-Saturation-Value (HSV) color histogram comparison and Speeded Up Robust Features (SURF) feature extraction together with the Fast Library for Approximate Nearest Neighbors (FLANN) double-checking matching. Qiong et al. [18] detected inter-frame forgery based on the histogram of oriented gradients (HOG) and motion energy image (MOI). 

Some studies tended to use deep learning methods in forgery detecting and locating but faced problems such as low accuracy, only detected forgery and some of them were forced to use labeled training sets as they used supervised learning. Long et al. [19] detected and localized frame duplicated frames in videos using a coarse-to-fine deep Convolution Neural Network (CNN) framework. This paper used the Siamese network with the ReSnet network to identify duplicated frames. Bakas and Naskar [20] detected frame insertion, duplication and deletion using a 3D convolutional neural network that used another CNN layer, which was used for temporal information extraction from videos. Li et al. [21] extracted features and localized abnormal points. In the extracting feature phase, the 2-D phase congruency of each frame was detected, since it was a good image characteristic. Then, the correlation between the neighboring frames was determined. In the second phase, the abnormal points were identified using a clustering algorithm (k-means). The normal and abnormal points were clustered into two categories.

The first video forgery type is multiple/double compression which occurs when a video is to be manipulated in compressed format [22,23]. The second type is region tampering which occurred by copying and pasting small parts of the frame at another location [24,25,26]. There is little attraction to the researchers for first and second types of inter-frame video forgeries. Table 1 summarizes the forgery type, feature method used, strengths and limitations of previously discussed studies. 

According to the previous problems, the main challenge is the manipulation of large videos. The tensor structure provides an excellent method for representing many kinds of highly correlated data such as videos. It is used in many applications as in [27,28,29]. Cheng et al. [8] discussed tensor data decomposition and its great influence on dimension reduction. Tensor data are routinely encountered in many fields such as genomics, image processing, finance and chemometrics. In Kountchev et al. [30] the advantages of third-order tensors and their application in video representation in multi-dimensional order were discussed. A third-order tensor was used to reduce the computational complexity. A new three-Dimensional Inverse Spectrum Pyramid (3D-ISP) approach was proposed for hierarchical third-order tensor decomposition. The tensors were transformed into 3D WalshHadamard spectrum space forms (WHT) that provided high dimensionality reduction.

## 3. Proposed Method

**Figure 1:**
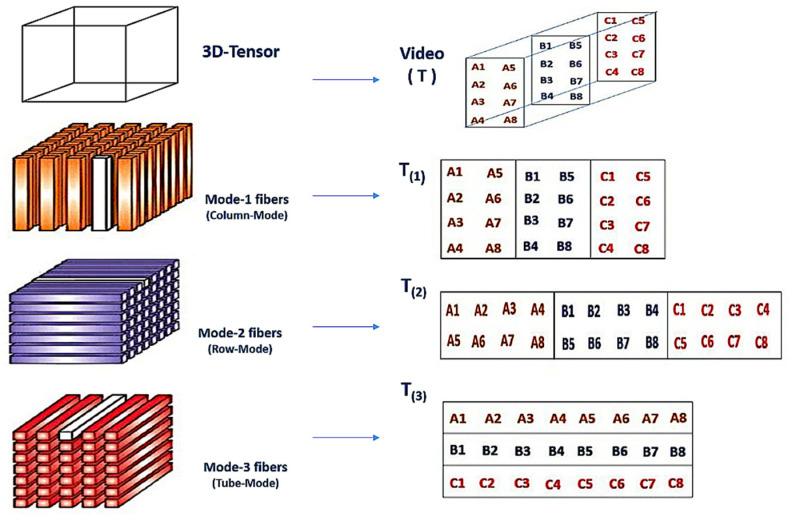
Third-order tensor construction and unfolding matrices.

The proposed method undergoes passive approaches for the detecting and locating of inter-frame video forgeries. However, instead of spatially comparing the whole pixel correlation through all successive frames, a group of tracing orthogonal features [31,32] is extracted from a third-order tensor representation of tube fiber geometrical frame construction and compared with its successive groups. Third-order tensor video construction, as depicted in Figure 1, is a representation of high dimensionality data with a multiway array structure. The three-way arrays of a third-order tensor are not called row vector and column vectors but are called tensor fibers. The tensor fiber is a one-way array with at least one subscript fixed. The fibers of a third-order tensor are vertical, horizontal and depth fibers that can be represented in three different modes. The vertical fibers of the third-order tensor are called column fibers (the column subscript is fixed) and the horizontal fibers are also known as row fibers (the row subscript is fixed). The depth is also called tube fiber (the row and column subscripts are fixed). 

In the proposed method, mode-3 fibers are used. Since tube fibers preserve the continuity of the spatial and temporal video scene together with its correlation characteristics, in addition, the tracing features extracted from third-order tensor representation achieve high dimensionality reduction and exact continuity measure [8].

**Figure 2:**
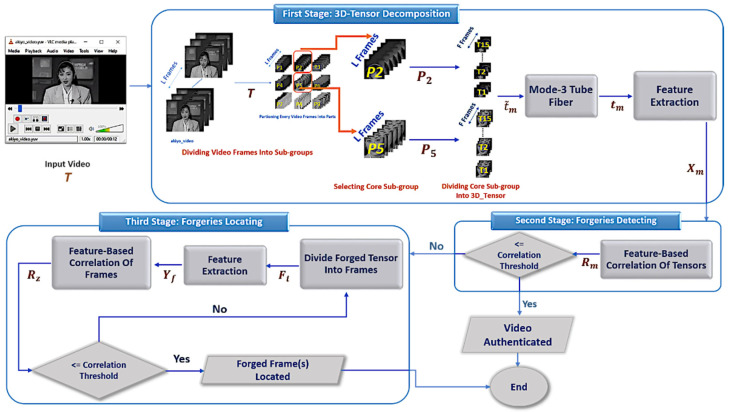
The proposed methodology.

The methodology of the proposed approach is illustrated in Figure 2. It consists of three successive phases: (i) Third-order tensor decomposition, (ii) Forgery detecting and (iii) Forgery locating. The next subsections present detailed explanations for each phase.

### 3.1. First Phase: 3D-Tensor Decomposition

This phase is used to geometrically construct a third-order video tensor representation. As mentioned earlier, the main contribution in this phase is the great accuracy and reduction in computations, especially when dealing with large videos. Table 2 indicates the abbreviation list of variables used in this paper. The steps are given in details as follows.

#### 3.1.1. Tube Fibers Representation

Consider an input video *T* consisting of *L* frames, each has a dimension of *H × W* pixels, where *H* and *W* represent the total number of rows and columns, respectively. The video sequence *T* is divided into equal sub-groups *P* each of length equals *L* frames, each sub-group *P* is represented by a number of third-order tensors (mode-3 (tube fiber)) that is used to represent the flow of video data, which is a vector defined by fixing the first two indices (row and column, respectively) and varying the third index (number of frames), Here the 3D tensor is not represented by all frames, but the core *P* of the video frames that are always changed in the video. Practically, only one core sub-group *P* is chosen for 3D tensor representation to test video authenticity. Now, the mathematical expression that describes the above explanation is Equation (1):

(1) $$T=\overset{N}{\underset{n=1}{\cup }}P_{n}$$

where *P_n_* is the *n*th sub-group *P*, and *N* is total number of sub-groups of the input video. After dividing the video into sub-groups, core sub-groups are selected to be represented by several 3D tensors ${\tilde{t}}_{m}$, as Equation (2):

(2) $$P_{n}=\underset{M}{\cup }{\tilde{t}}_{m}(i,j,k):i=\{0,1,2,\ldots h\},j=\{0,1,2,\ldots w\},k=\{0,1,2,\ldots F\}$$

where *F < L*, is the total number of frames of each 3D-tensor ${\tilde{t}}_{m}$, as *F* decreases the accuracy of detecting forged frames increases, and vice versa. However, for the proposed techniques, it should not decrease by 10 frames or increase by 30 frames to get high detection accuracy, low computational complexity and to help in locating inter-frame forgeries as will be seen in the experimental results section. Finally, *w* and *h* are the selected number of columns and rows *t_m_*, where: *h < H*, and *w < W* and *m* = {*1,2, …, M*}, *M* is the number of all 3D tensors.

Referring to Figure 1, each ${\tilde{t}}_{m}$ is represented mathematically by a mode-3 tube 2D matrix as Equation (3):

(3) $$\begin{array}{l} t_{m}=I\left(F,h,w\right)=\\ \left[\begin{array}{c} I_{1}\left(1,1\right)\ldots I_{1}\left(h,1\right)I_{1}\left(1,2\right)\ldots I_{1}\left(1,w\right)\ldots I_{1}\left(h,w\right)\\ I_{2}\left(1,1\right)\ldots I_{2}\left(h,1\right)I_{2}\left(1,2\right)\ldots I_{2}\left(1,w\right)\ldots I_{2}\left(h,w\right)\\ .\\ .\\ .\\ I_{F}\left(1,1\right)\ldots I_{F}\left(h,1\right)I_{F}\left(1,2\right)\ldots I_{F}\left(1,w\right)\ldots I_{F}\left(h,w\right) \end{array}\right] \end{array}$$

For example, if a total video container matrix *T* has dimensions of (192 × 192 pixels) × 300 frames, it can be divided into a total of nine *P* sub-groups, each with dimensions of (64 × 64 pixels) × 300 frame. The most important sub-groups can be chosen to be divided into a group of third-order tensors which are represented as a 2D matrix as in Equation (3) with dimensions of 20 × 4096 pixels. Here, it can be noted that the dimensions division process is arbitrary and corresponds to the nature of the scene of the suspected video.

#### 3.1.2. Feature Extraction

Feature extraction is an important step for reducing data dimensionality, computational time and complexity. Each 2D matrix *t_m_* is processed for feature extraction. There are many feature extraction methods used in forgery detecting and locating. Based on the previous studies, the three most effective methods used for extracting good features to trace are: Harris [33,34], Gray Level Co-occurrence Matrix (GLCM) [6] and Singular Value Decomposition (SVD) [22], In this paper, each of which is applied for 2D matrix, tested and compared to obtain the best combination.

##### Harris Feature Extraction

In this step, Harris feature extraction is applied for each 2D matrix *t_m_* as in Equation (3). Different detectors of the interest points were suggested and used based on the application field. The Harris detector, which is the fast, robust and rotation invariant, is commonly used in many computer vision applications that use the autocorrelation function to determine locations where the signal changes in one or two directions occur as in [33]. The concept behind the algorithm for Harris corners is that the intensity of the image will change significantly in several corner directions, while the intensity of the image will change significantly in a corner some direction along the edge and this phenomenon can be formulated by studying the changes in intensity resulting from local window shifts. The intensity of the image can change greatly around a corner point when the window is rotated in an arbitrary direction. At approximately an edge point, the intensity of the image will greatly change when the window is rotated in the perpendicular direction. Following this theory, the Harris detector uses a second-order moment matrix as the basis of its corner decisions. Unless otherwise specified, all corner points and edge points identified by the Harris corner detector refer to Harris corner interest points as in [34].

Harris feature extraction is applied for each tensor *t_m_* included in each core sub-group *P*. Therefore, the autocorrelation matrix *M* for a given third-order tensor *t_m_* at point *(x, y)* can be calculated as in Equation (4):

(4) $$M\left(x,y\right)=\sum_{x,y}W\left(x,y\right)\left[\begin{array}{cc} t_{x}^{2}\left(x,y\right) & t_{x}t_{y}\left(x,y\right)\\ t_{x}t_{y}\left(x,y\right) & t_{y}^{2}\left(x,y\right) \end{array}\right]$$

where *t_x_* and *t_y_* are pixel intensity respective derivatives in the *x* and *y* directions at point *(x, y)*. That is,

(5) $$t_{x}=t⊗\left[-1,0,1\right]\approx \partial t/\partial x$$

(6) $$t_{y}=t⊗{\left[-1,0,1\right]}^{T}\approx \partial t/\partial y.$$

where the operator ⊗ represents convolution. The off-diagonal entries are the product of *t_x_* and *t_y_*, while the diagonal entries are the squares of the respective derivatives and *t* is the element of *t_m_*. *W(x, y)* can be uniform in the weighting function, but is more generally an isotropic and σ represents standard deviation. Circular Gaussian as in Equation (7):

(7) $$W\left(x,y\right)=g\left(x,y,\sigma \right)=\frac{1}{2\pi \sigma^{2}}\exp\left(-\frac{x^{2}+y^{2}}{2\sigma^{2}}\right)$$

This gives greater weight to those values close to a local region’s center. Let α and *β* be the *M(x, y)* eigenvalues. These values provide a quantitative description of how the measure of autocorrelation changes its main curvatures in spatially. The image regions can be split into three groups according to the autocorrelation matrix eigenvalues: plain regions, edges, and corners. Note that the σ*β* product is sensitive to corners, while the σ + *β* sum is sensitive to both edges and corners. In addition, the trace and the determinant of a general diagonalizable matrix agree with the product and the sum of its eigenvalues:

(8) $$Tr\left(M(x,y)\right)=\alpha +\beta =t_{x}^{2}\left(x,y\right)+t_{y}^{2}\left(x,y\right)$$

(9) $$Det\left(M\left(x,y\right)\right)=\;\alpha \beta \;=t_{x}^{2}\left(x,y\right)\cdot t_{y}^{2}\left(x,y\right)-{\left(t_{x}t_{y}\left(x,y\right)\right)}^{2}$$

Using *Tr* (*M*(*x, y*)) and *Det* (*M*(*x, y*)) to determine the corner response is attractive because it prevents the need for explicit decomposition of the *M*(*x, y*) eigenvalue. The corner response is calculated using Equation (10):

(10) $$Corn\left(x,y\right)=Det\left(M\left(x,y\right)\right)-K.Tr^{2}\left(M\left(x,y\right)\right)=\sigma \beta -K.{\left(\sigma +\beta \right)}^{2}$$

where *K* is an empirically selected scalar value out of the range value (0.04, …, 0.16). Corner points have high positive eigenvalues and thus a large response to the Harris measure. Thus, corner points that are greater than a specified threshold are recognized as local maxima of the Harris measure response:

(11) $$\begin{array}{l} \left\{\left(x_{c},y_{c}\right)\right\}=\\ \{\left(x_{c},y_{c}\right)|Corn\left(x_{c},y_{c}\right)>Corn\left(x_{i},y_{i}\right),\forall Corn\left(x_{i},y_{i}\right)\in W\left(x_{c},y_{c}\right),Corn\left(x_{c},y_{c}\right)>t_{th}\} \end{array}$$

where {(*x_c_, y_c_*)} is the corner point set, *Corn*(*x_c_, y_c_*) is the Harris measure response computed at point (*x, y*), *W*(*x_c_, y_c_*) is an 8-neighbor set centered around point (*x_c_, y_c_*) and *t_th_* is a specified threshold. Obviously, the number of Harris corner points identified depends on the threshold *t_th_* [34].

##### GLCM Feature Extraction

Another different method for feature extraction is applied to improve the results of the Harris feature. Each sub-tube matrix p is processed for GLCM feature extraction. The Gray Level Co-occurrence Matrix (GLCM) is a method of texture feature extraction that is used effectively in various problems of image processing, such as segmentation, image recognition, classification, retrieval and texture analysis as in [6]. The GLCM method is used for feature extraction from video frames after which these texture features are subjected to correlation. GLCM is a statistical measurement of a second order (between two pixels or two pixels subgroups in an image). The non-normalized frequencies of co-occurrence can be interpreted as a function of angle and distance as follows. Four GLCMs for *θ* = 90° are constructed. Ninety degrees as video frames are arranged in tube tensor as Equation (12).

(12) $$t_{90^{{}^{\circ}},d}\left(a,b\right)=\left|\left\{\left(\left(k,l\right),\left(m,n\right)\right):\left|k-m\right|=d,l=n\right\}\right|$$

where (*k, l*) and (*m, n*) express the locations of pixels with gray levels *a* and *b. a, b* represent the gray levels of pixel within a frame window separated by distance *d* and *|{···}|* represents set cardinality.

##### SVD Feature Extraction

Due to the nature of motions in video scenes, the required features must satisfy certain specifications. These features must provide stability, scaling properties and rotation invariance, to help trace those features through entire sub-tubes. SVD is a matrix factorization that has algebraic and geometric invariant properties. It has the ability to extract unique features for an image, which form a steady representation of image blocks. It has proven a great performance results in different applications [22,35]. 

SVD feature extraction is the method of robust and accurate decomposition of the orthogonal matrix. It is becoming increasingly common in the field of signal processing because of conceptual SVD and stability reasons. Image processing is an attractive algebraic transformation. 

In a minimally square sense, the SVD is the ideal matrix decomposition that stores the full signal energy into as few coefficients as possible. It is an effective and stable method of dividing the matrix into a set of linearly independent components, each with a contribution of its energy. It is a numerical method used in numerical analysis to diagonalize matrices. Due to its endless advantages such as maximum energy packing which is usually used in compression, ability to manipulate the image based on two distinctive subspaces of data and noise subspaces, it is an attractive algebraic transformation for image processing, which is commonly used in noise filtering and is also utilized in watermarking applications. 

In this paper, the SVD algorithm is deployed to third-order tensor. For each *t_m_*, a singular value obtains the feature vectors of each part via SVD, which is given by Equation (13):

(13) $$t_{m}=UX_{m}V^{T}$$

*U* and *V^T^* are the unitary matrices, and *X_m_* is the singular value of *t_m_* which is a diagonal matrix. The one-dimensional vector is formed from the diagonal elements of *t_m_*, and the vector can be expressed as *X_m_* = {*x_m1_*,_…_, *x_mQ_*}*. X_m_* a feature vector of *t_m_*. 

### 3.2. Second Phase: Forgery Detecting

#### 3.2.1. Features-Based Correlation of Tensors

Here, the autocorrelation between consecutive tensors features is calculated. For example, after extracting SVD feature vector *X_m_* for each mode-3 tube 2 D- matrix, the correlation coefficient between every two consecutive feature vectors is calculated using the standard Pearson correlation [36] as in Equation (14):

(14) $$R_{m}=\frac{\sum_{t}\left(x_{m}\left(t\right)-\bar{x_{m}}\right)\times \left(x_{m+1}\left(t\right)-\bar{x_{m+1}}\right)}{\sqrt{\sum_{t}{\left(x_{m}\left(t\right)-\bar{x_{m}}\right)}^{2}\times \sum_{t}{\left(x_{m+1}\left(t\right)-\bar{x_{m+1}}\right)}^{2}}}$$

where $R_{m}$ is the correlation between each two consecutive feature vectors of *t_m_* and *t_m_ + 1* tensors. Here, $X_{m}\left(t\right)$ is the *m*th SVD feature of the *t_m_* tensor and $\bar{X_{m}}$ represents the average of all SVD features of the mth tensor. This is repeated for all chosen *P* of the input video. For example, if a video consists of 300 frames, it is divided into several *P* according to its size, the chosen core *P* are divided into tensors and so be 15 tensors, each of which contains 20 frames. The correlation is calculated between every consecutive pairs of these 15 tensors to get 14 correlation values. These values are statistically averaged to get an average value of the correlation among tensors. Hence, a threshold value is calculated based on the obtained statistics and is used to detect video forgery. Thresholds vary in correspondence to the nature of each video. Using Chebyshev’s inequality [37], this threshold is computed as follow:

(15) Threshold=μ−m·σ

where *µ* and *σ* are the mean and the standard deviation, respectively, of correlation distribution *R_i_* values of the total adjacent *m* tensors. Their mathematical representations are as follows:

(16) $$\mu =\frac{\sum_{i=1}^{m-1}R_{i}}{m-1}$$

(17) $$\sigma =\sqrt{\frac{\sum_{i=1}^{m-1}{\left(R_{i}-\mu \right)}^{2}}{m-1}}$$

For unknown data distribution, the lower bound for the threshold within a group of adjacent tensors can be determined by applying Chebyshev’s inequality. The correlation value computed from Equation (14) is compared with the computed threshold to define the type of forgery as insertion or deletion. Algorithm 1 illustrates the procedure of detecting.
**Algorithm 1** Forgery Type Determination.   **Input**: Correlation values *Rm* where m = 1: M and *Threshold*. (14)–(15)   **Output:** Forgery type.1.   **Begin**2.     **for**
$R_{m}$ where m = 1: M **do**3.        **if**
$R_{m}$ & $R_{m+1}$ <= *Threshold*
**then**4.           Forgery type is insertion5.        **else if**
$R_{m}$ <= *Threshold*
**then**6.           Divide tensors with suspected values into Sub-Frames.7.          **if** two suspected points are found **then**8.              Forgery type is insertion9.           **else**10.             Forgery type is deletion11.           **end**12.        **else**13.           No forgery (video is original)14.        **end**15.     **end**16.  **end**

#### 3.2.2. Insertion Forgery Detecting

**Figure 3:**
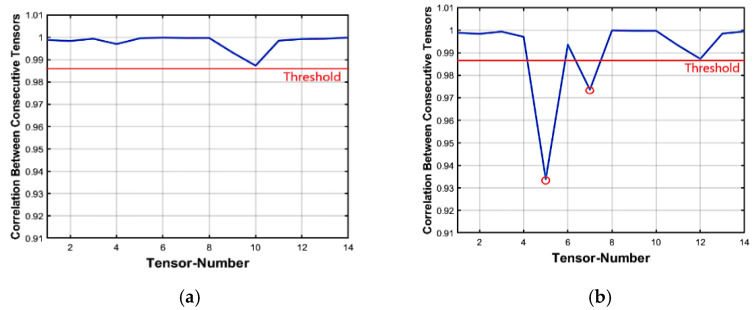
Inter-tensor correlation distribution analysis. (**a**) Original video Inter-tensor correlation distribution and (**b**) Forged video Inter-tensor correlation distribution (Insertion attack).

For more illustrations, let us consider a practical implementation for Algorithm 1. The tensor correlation distribution analysis of the original foreman video dataset is shown in Figure 3a. The video consists of 300 frames and is divided into 15 tensors and each tensor contains 20 frames. Figure 3b depicts the frame insertion forgery correlation distribution analysis after inserting 40 frames from external video starting as mentioned earlier. Now, considering Figure 3b, the two abnormal tensors-correlation drops comparing with the threshold value, (Algorithm 1—step 7) represent the start and the end forged tensors, respectively. These two abnormal points correspond to point 5 (which indicates correlation between the 5th and 6th tensors) and point 7 (which indicates correlation between the 7th and 8th tensors). This verifies that there are forged frames in tensors number 5, 6, and 7 respectively.

#### 3.2.3. Deletion Forgery Detecting

**Figure 4:**
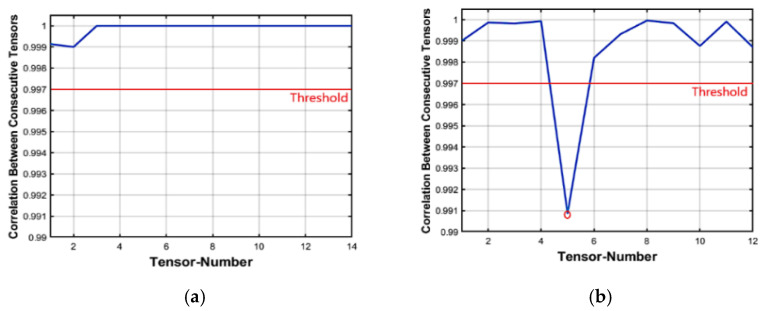
Inter-tensor correlation distribution analysis. (**a**) Original video Inter-tensor correlation distribution and (**b**) Forged video Inter-tensor correlation distribution (Deletion attack).

To detect the frame deletion forgery case, the proposed method is applied to the forged dataset. For testing, we made 50 forged datasets for the deletion case. The correlation distribution analysis for the foreman dataset is shown in Figure 4a. Recall that the original video consists of 300 frames divided into 15 tensors at each part and each tensor contains 20 frames. Figure 4b indicates the frame deletion forgeries correlation distribution analysis in the forged video, 30 frames deleted from this video starting from frame number 100 ended at frame number 130. As presented in Figure 4b, one abnormal point is found at 5 (Algorithm 1—step 10) which indicates a correlation between the 5th and 6th tensors. This shows that there is a forgery attack in tensors 5, 6, and 7. 

### 3.3. Third Phase: Forgery Locating

Recalling the proposed methodology, Figure 2, this phase is applied only if the video is detected as forged. The purpose of this phase is to locate the forged frames. Next, its steps are explained in detail.

#### 3.3.1. Tensors Analysis

In the case of detecting forgery between two consecutive tensors, one tensor before and one tensor after are invoked, all these tensors are analyzed as frames (in our example 20 frames per tensor) to locate forgery in the video. The extracted frames are denoted by *Fi* (*i* = *1, 2, …, F*). The feature vectors of each frame via SVD are obtained, which are given by:

(18) $$S_{f}=UY_{f}V^{T}$$

*S_f_* is SVD matrix of each frame in 3D-tensor, *Y_f_* = {*Y_f1_, …, Y_fB_*} is one-dimensional vector as a feature of *f_l_* and *Y_f1_* and *Y_fB_* are first and last feature values. 

#### 3.3.2. Features-Based Correlation of Frames

After calculating singular values for each sub-frame in selected forged tensors, the correlation coefficient between every two consecutive sub-frames is computed. According to the correlation values, the threshold is determined to localize the forgery in the video. The same equation is applied in but between every consecutive frame as:

(19) $$R_{z}=\frac{\sum_{f}\left(Y_{z}\left(f\right)-\bar{Y_{z}}\right)\times \left(Y_{z+1}\left(f\right)-\bar{Y_{z+1}}\right)}{\sqrt{\sum_{t}{\left(Y_{z}\left(f\right)-\bar{Y_{z}}\right)}^{2}\times \sum_{t}{\left(Y_{z+1}\left(f\right)-\bar{Y_{z+1}}\right)}^{2}}}$$

where $R_{z}$ denotes the correlation between the *f*th and (*f + 1*)th subframes, $Y_{z}\left(f\right)$ refers to the *z*th SVD feature of the *z*th Sub-frames, and $\bar{Y_{z}}$ refers to all SVD features means of the *z*th sub-frames. For example, if forgery is detected in tensors 5, 6 and 7, then these tensors are divided into frames from 100 to 160 and correlation is calculated between these frames to locate the position of forgery. According to the correlation values, the threshold is determined using the same Chebyshev’s inequality [37] except that the mean and the standard deviation Equations (16)–(17) are calculated for the internal frames in each ${\tilde{t}}_{m}$. The same procedure is used to localize the forgery in the video.

#### 3.3.3. Locating Forgeries

##### Insertion Forgeries

**Figure 5:**
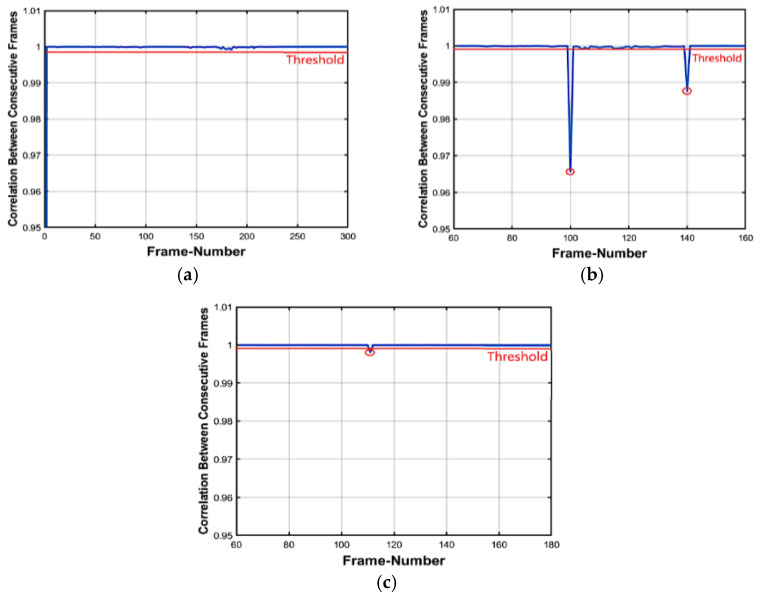
Inter-frame of foreman video sequence Correlation distribution: (**a**) Original video and (**b**) Forged video (Insertion attack) (**c**) Forged video (Deletion attack).

Forgeries are simply localized from abnormal values in the inter tensor correlation distribution. However, for locating refinement, an inter-frame correlation distribution is applied. The distribution analysis for the foreman original video is shown in Figure 5a, which indicates that the correlation between frames is very high. Figure 5b shows the frame insertion forgeries correlation distribution analysis in the foreman video sequence. Forty frames from a foreign video were inserted starting at frame number 101 and ending at frame number 140 and two abnormal points were detected: the first point indicated the first inserted frame and the other indicated the last inserted frame. This is the final step in which we can localize the forged inserted frames.

##### Deletion Forgeries

Figure 5c shows the frame deletion forgeries inter-frame correlation distribution analysis in the video sequence. More analysis is performed starting from frame number 60 to frame number 160 and the results in the localization of 30 missing frames starting from frame number 111 were deleted. This is the final step in which we can localize the forged deleted frames. Algorithm 2 illustrates the proposed scheme of inter-tensor and inter-frame correlation to localize the insertion and deletion forgeries in videos.
**Algorithm 2** Forgery Location Determination. **Input**: Correlation values *Rm* where *m* = 1: *M*, *Threshold*, t which is tensor number. **Output:** Number of inserted or deleted Forged frames.1. **begin**2.  **for**
$R_{m}$ where m = 1:M **do**3.   **if** Forgery is detected at $R_{m}$ & $R_{m+1}$
**then**4.    Forgery type is insertion.5.    Divide tensors whose numbers are t − 1, t, t + 1, t + 2 into frames (from s to n).6.    Compute correlation between every two consecutive frames in $R_{z}$.7.     **for**
$R_{z}$ where z = 1:n-1 **do**8.      **if** Two suspected values are found **then**9.      Forgery location determined10.     **end**11.   **else if** forgery is detected at $R_{m}$
**then**12.     Repeat steps 5, 6.13.     **if** two suspected values are found **then**14.      Forgery type is insertion and forgery determined15.     **else if** one suspected value is found **then**16.      Forgery type is deletion and forgery determined17.     **end**18.   **else**19.    No forgery20.   **end**21.  **end**22. **end**

## 4. Experimental Results and Discussion

To evaluate the performance of the proposed scheme, a MATLAB computer simulation program (R2018a, MathWorks, Natick, MA, USA) was developed for testing and validating several experiments. The computer configuration used in these experiments is described as follows: CPU: Intel(R) core (TM) i7-9750H CPU @2.60 GHZ (Lenovo, Beijing, China); Memory size: 16 GB RAM; OS: Microsoft Windows 10 (Microsoft, Redmond, WA, USA); the Coding: MATLAB R2018a;. The next subsections explain the tested dataset, the standard evaluation parameters. Finally, comparisons and discussion are introduced.

### 4.1. Tested Dataset Description

Experiments on the proposed scheme are performed with a standard dataset consisting of eighteen video clips with a frame rate of 30 frames per second (fps), from the TRACE library, where each YUV sequence is either in Quarter Common Intermediate Format (QCIF) which is (176 × 144) format or Intermediate Format (CIF) which is (352 × 288) format [38]. The tested dataset contains videos with static backgrounds, slow-motion backgrounds, fast-moving (single or multiple) foreground objects, zoom in and zoom out. Table 3 summarizes the characteristics of the tested datasets.

Manual forgeries are performed for frame insertion and deletion attacks on the above dataset. Videos are made using the ffmpeg tool which provides command-line or programmatic access to video and audio processing. The original video is first decomposed into individual frames, and then the forgery is performed by inserting or removing frames. In this paper, both forgery attack experiments are tested against small and large numbers of forged frames to test the robustness of the proposed scheme. Forged videos are created starting with 10 forged frames up to 50 frames. Forged videos are created using the Audio Video Interleave (AVI) extension in MATLAB R2018a and eventually, the forged videos are translated into the .YUV extension.

### 4.2. Evaluation Standards

To evaluate the validity of the scheme, three performance indices are considered: precision, recall and F1 score [39,40,41] which are computed as follows:

(20) $$Precision=\frac{TP}{TP+FP}$$

(21) $$Recall=\frac{TP}{TP+FN}$$

(22) $$F1\;score=\frac{2\times Precision\times Recall}{Precision\times Recall}$$

where *TP* is the true positive number which means that the forged video was detected as forged, *TN* is the true negative number which means that the original video was detected as original, *FP* is the number of false positive which means that the original video was detected as forged and *FN* is the number of false negatives which means that forged videos were detected as the original. 

### 4.3. Computational Complexity Analysis

The proposed technique offers a great advantage of speeding up the detecting and locating process since it offers a great opportunity for parallel processing for different tensors at the same time instead of consecutive frame processing compared with state-of-the-art methods. This advantage has a great influence on the total time needed for forgery detecting and locating as will be discussed later. However, tensor size is linearly proportional to the number of computations.

**Figure 6:**
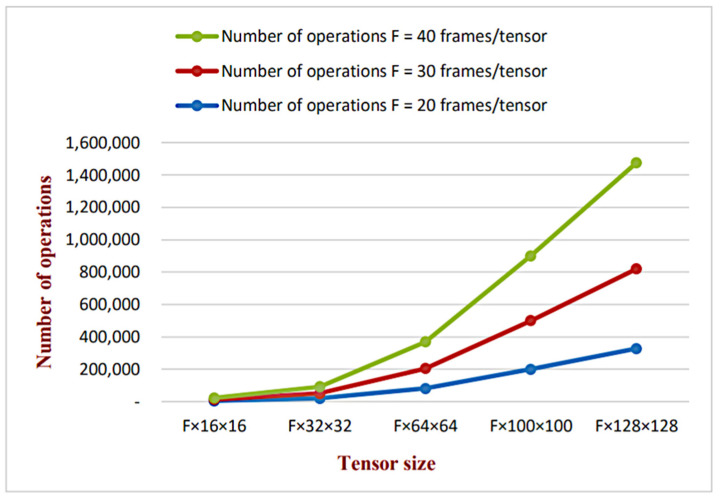
The increase in total number of operations against the increase in tensor size.

Table 4 illustrates the relation between tensor size and the total number of operations needed in the detecting and locating process. Through our simulation, 20 frames in every tensor are selected as it has a great reduction in the total number of operations while providing high detection accuracy. This relation also is graphically illustrated in Figure 6. The total number of operations per tensor is calculated using the MATLAB R2018a counting operations function. Compared with state-of-the-art methods, most of them calculate the correlation between the whole frame’s pixels/frame’s features of different frames along the video sequence. However, no previous data about computational complexity was mentioned before in state-of-the-art methods since it mainly depends on the programmer’s skills. It can be obviously seen that the proposed tensor structure is proven to provide a high reduction in the total number computations since a limited number of tensors of small size are needed for detecting and locating process instead of dealing with whole sequences and the entire frames/features.

## 5. Comparisons and Discussion

In this section, the proposed scheme is applied to the eighteen datasets depicted in Table 3, and their forged versions. Tested against two types of forgery: insertion and deletion. The comparison results of applying three methods of feature extraction: Harris feature extraction, GLCM feature extraction and SVD, on a maximum of hundred forged videos for insertion and deletion cases, are introduced and discussed. Each of them influences the results as introduced in the following subsections.

### 5.1. Insertion Forgery

For testing forgery attack detecting and locating, several experiments were conducted to trace the performance accuracy of the proposed scheme against the increase/decrease in the number of forged frames. Table 5 shows and compares the precision of the detecting and locating phases. The proposed scheme shows a noticeable enhancement when applying the SVD feature extraction method. Precision up to 96% in the detection phase is reached and 99% in localization capability. These results reflect the stability, scaling property and geometric invariance property of the SVD feature extraction method.

**Figure 7:**
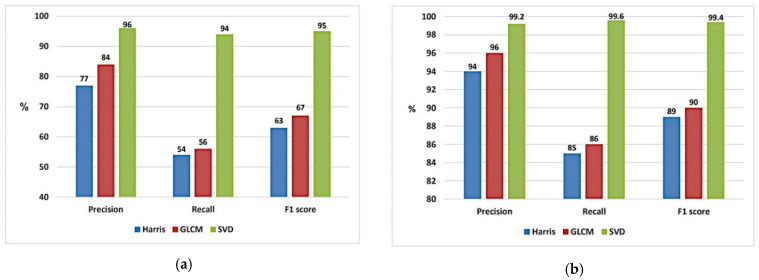
Performance chart of three different feature extraction techniques used for insertion forgery cases. (**a**) Insertion detecting phase and (**b**) Insertion locating phase.

As shown in Table 5, the greater the number of frames inserted from the external video, the faster the forgery position is determined because this increase of forged frames causes a significant change in the content of the video. The charts in Figure 7a,b visually summarize the results of Table 5. It visually points out the superiority of the SVD feature extraction method in both detecting and locating phases, and it has the best results in terms of precision, recall and F1 score. For more robust investigations, the proposed scheme is tested against the increase in the number of frames inserted into the original videos. 

**Figure 8:**
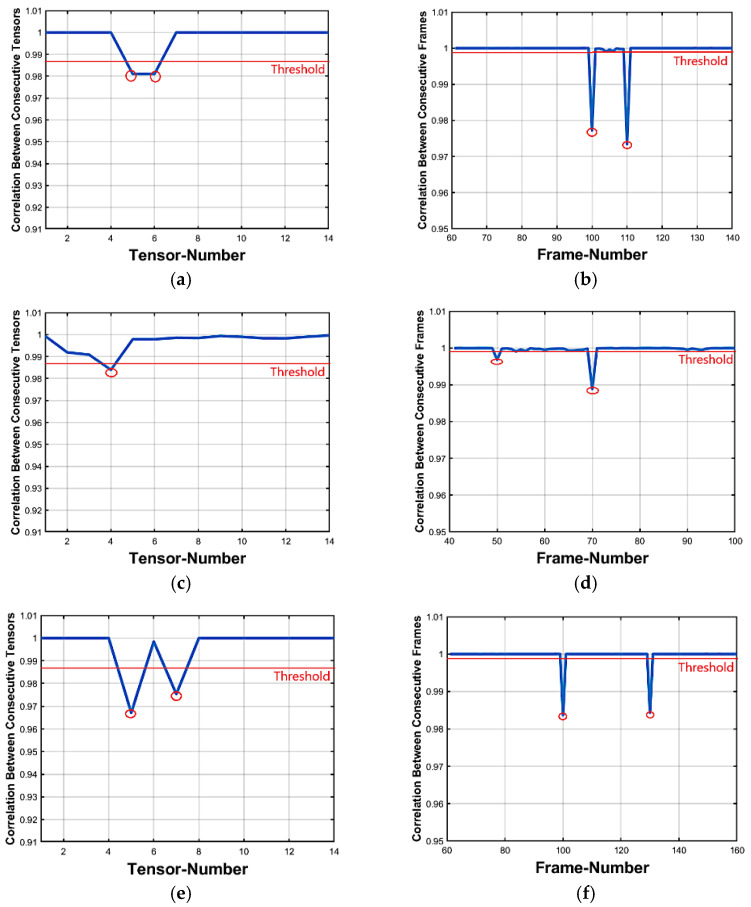

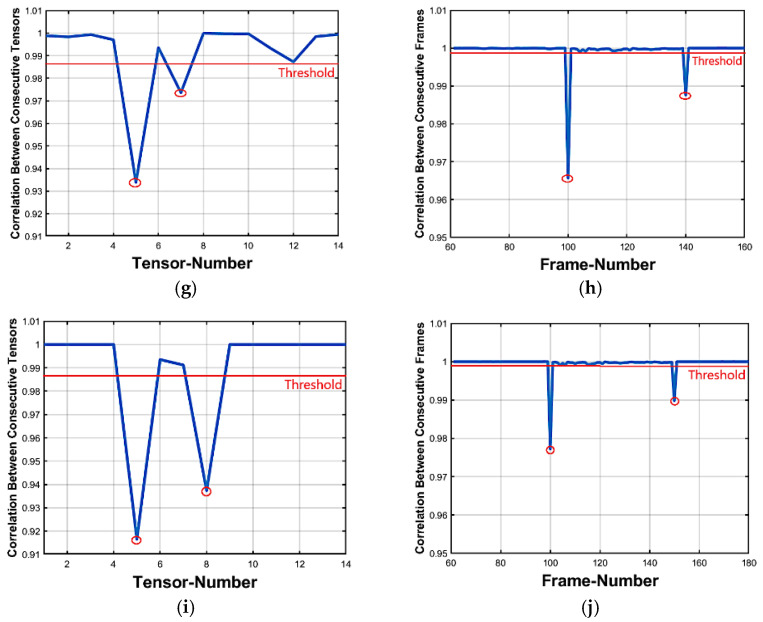
(**a**,**c**,**e**,**g**,**i**) insertion forgery detecting and (**b**,**d**,**f**,**h**,**j**) insertion forgery locating of 10, 20, 30, 40 and 50 forged frames respectively.

Figure 8 shows the detecting and locating results for five different videos under different numbers of inserted frames. The left side of this figure shows the inter tensor correlation figures that detect the existence of forgery and at this level, there are almost two or sometimes one abnormal value that expresses insertion forgery while the right side accurately localizes the number of inserted foreign frames. This right side indicates that two abnormal values indicate the start and the end of forgery in videos.

### 5.2. Deletion Forgery

**Figure 9:**
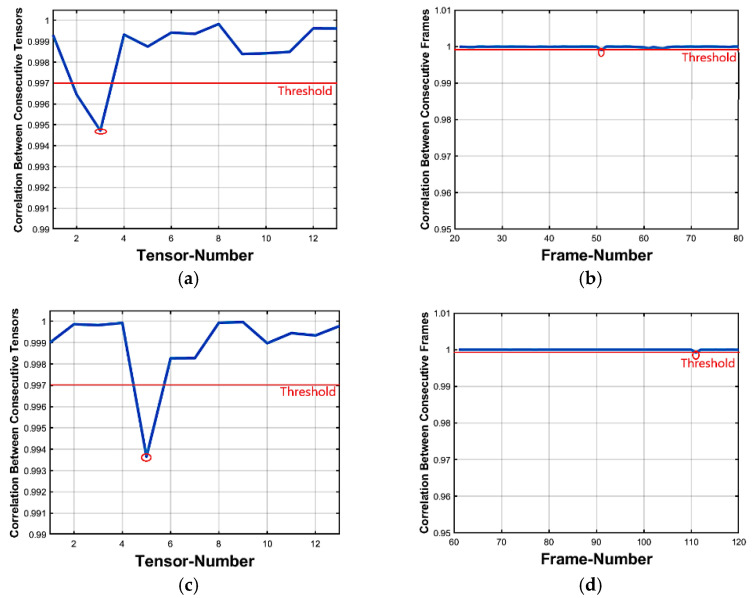

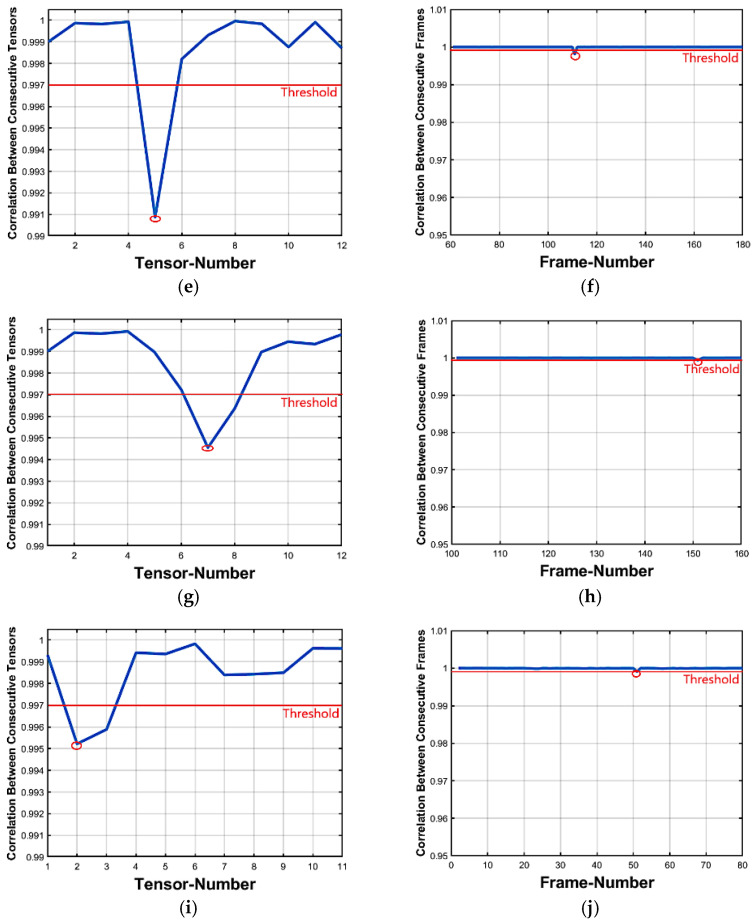
(**a**,**c**,**e**,**g**,**i**) deletion forgery detecting and (**b**,**d**,**f**,**h**,**j**) deletion forgery locating of 10, 20, 30, 40 and 50 forged frames respectively.

The proposed scheme is tested and evaluated against the detecting and locating of deletion forgeries with different cases of deleted numbers of frames. As mentioned before, the SVD feature extraction method is used in deletion attacks as it achieves efficient results in insertion attacks. Table 6 shows the results of detecting and locating these different cases. It is very difficult to detect and localize deletion forgeries for fewer than 10 frames in the video as the changes in it are very small. However, the proposed scheme shows large robustness in detecting and locating against the increase in the number of deleted frames (up to 50 frames). Precision up to 92% in the detecting phase is reached and 98.4% in the locating phase. Figure 9 illustrates results for five different videos under different numbers of deleted frames. The left side of this figure shows the inter-tensor correlation figures that detect the deletion forgery existence and in this, there is only one abnormal point that always indicates the forgery, while the right side accurately localizes the position of the deleted forged frames and in this right level there is only one point that indicates the position of the forgery.

### 5.3. Comparison with State-of-the-Art

**Figure 10:**
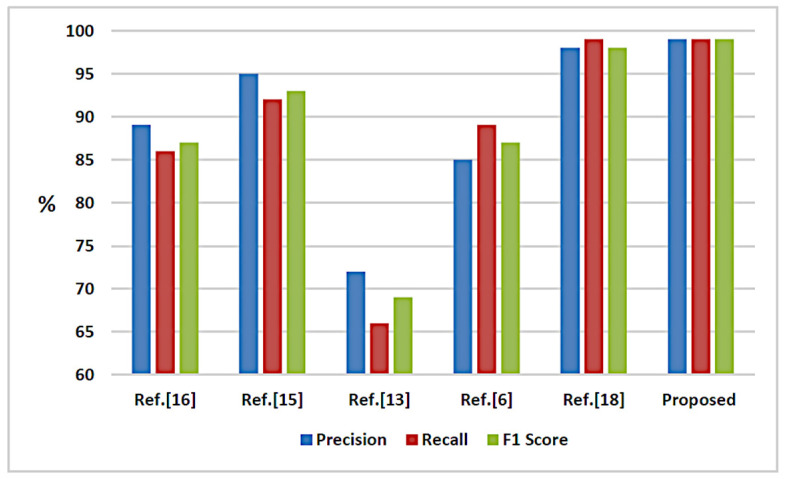
Performance chart of proposed approach compared with other related methods.

Comparison with the state-of-the-art is provided in order to compare the proposed scheme performance with different methods. We tested all methods on the same dataset. Table 7 summarizes the comparative results for both types of forgery among the recent techniques and the proposed one. The overall precision, recall and F1 score of the proposed methods are 99%, 95% and 96% respectively which shows superiority compared with published methods. Figure 10 illustrates these results.

The method proposed by Yu et al. [13] detected and localized frame deletion forgeries only. The scheme proposed by Aghamaleki and Behrad [16] is applicable to frame insertion and deletion forgery in low accuracy. Zhang et al. [15]’s scheme can detect frame insertion/deletion video forgeries for still background videos. Bakas et al. [6] proposed a method that can detect frame insertion, deletion and duplication forgeries for still background, as well as dynamic background videos but the comparison was performed with insertion and deletion results. The scheme proposed by Qiong et al. [18] is for insertion, deletion and duplication cases but it took many computations and failed in detecting frame deletion in silent scenes.

The proposed method of this paper can detect insertion and deletion forgeries for a still background as well as dynamic background videos. The proposed method offers high accuracy in respect of the achieved precision at a minimum number of features compared with previous works. 

Recalling that the proposed tensor geometric structure provides a high reduction in computational time due to the small size of tensors and the possibility of processing tensors in a parallel manner rather than the consecutive approaches used in the state-of-the-art. However, it is difficult to compare experimental time with the state-of-the-art methods although they used the same dataset since different computer configurations together with different programmers’ skills are deployed. In this paper, based on the previously mentioned computer configurations used in these experiments, the average computation time per tensor is less than 2.2 s. Third-order tensor representation together with a good feature extraction method offered this great reduction. Considering the average computations time for previous methods [6,17,42], although different computer configurations were used, the proposed method clearly outperforms these methods, since a limited number of tensors is used in the investigation process rather than the other methods that exploit the whole frame’s pixels/frame’s features. Table 8 illustrates the total time needed for forged frames detecting and locating. It can be noted that as the number of inserted forged frames increases, the total time increases since more computations for tensors are needed, while as the number of deleted frames increases, the total time decreases since the number of frames decreases.

## 6. Conclusions

Videos are linear groups of highly correlated data that consume time and computational complexity. Recently, the most common methods for video compression represents such data on the basis of a geometric tensor representation. This paper proposed a low computational complexity scheme based on tensor representation and orthogonal tracing feature algorithms for detecting and locating insertion and deletion forgery in videos. Three different common tracing features were tested, evaluated, and compared to choose the outperforming one. Experiments and comparisons showed the superiority of SVD tube-fiber tensor construction in detecting and locating these two types of video forgeries. Different datasets of different characteristics were examined, and the proposed scheme was tested against the increase in the forged frame number. The proposed method performed efficiently for static as well as dynamic videos, quick-moving foreground items (single or multiple), zooming in and zooming out datasets. Experimental results showed that the proposed approach obtains effective accuracy with a high precision value of up to 99% and a reduction in time and computational complexity. Future research in this direction is still open, and it will include enhancing the detecting and locating process for more types of attacks.

## Figures and Tables

**Table 1:** Video forgery detecting methods.

References	Forgery Type	Feature Method Used	Strengths	Limitations
[10]	Frame duplication	Similarity between SVD features vector of each frame.	High accuracy in detecting forgery	Failed in detecting other types of forgery such as insertion or reshuffling.
[11]	Frame duplication	Correlation between the successive frames.	Detected and localized frame duplication in higher accuracy.	Failed when frame duplication was performed in a different order.
[12]	Frame deletion	Sequence of average residual of P-frames (SARP) and its time- and frequency-domain features.	Was very effective with the detecting.	Worked with fixed GOP only.
[13]	Frame deletion	Magnitude variation in prediction residual and intra macro blocks number.	Worked stably under various configurations.	Failed if the number of deleted frames was very small.
[14]	Frame insertion and deletion.	Correlation coefficients of gray values.	Efficient in classifying original videos and forgeries.	Worked with still background datasets.
[15]	Frame insertion and deletion.	Quotients of correlation coefficients between (LBPs) coded frames.	High detecting accuracy and low computational complexity.	Detected only if forgeries exist but cannot distinguish frame insertion and deletion.
[16]	Frame insertion and deletion.	Quantization error in residual errors of P-MB in P frames.	Effective detecting.	Not suitable for videos with a low compression ratio.
[6]	Frame insertion, deletion and duplication.	Correlation between the Haralick coded frame.	Worked efficiently for static as well as dynamic videos.	Not able to detect other types of forgery such as frame reshuffling and replacement.
[17]	Frame insertion, deletion and duplication.	HSV color histogram comparison and SURF.	Was efficient and accurate in terms of forgery identification and locating.	Failed to detect inter-frame video with many shots.
[18]	Frame insertion, deletion and duplication.	HOG and MOI.	Was efficient in insertion and duplication.	Failed to detect frame deletion in silent scenes.
[19]	Frame duplication.	An I3D network and a Siamese network were used.	Detected frame duplication in an effective method.	Compression might decrease the accuracy and failed to detect frame deletion forgery.
[20]	Frame insertion, deletion and duplication.	(3D-CNN) is used for detecting the inter-frame video forgery.	Detected inter-frame video forgeries for static as well as dynamic single-shot videos.	Failed in localization of forgeries and detecting of multiple video shot forgeries.
[21]	Frame insertion, deletion and duplication.	Correlation between 2-D phase congruency of successive frames.	Localized the tampered positions efficiently.	Failed in distinguishing whether the inserted frames are copied from the same video or not.
[22]	Multiple/double compression	Pixel estimation and double compression statistics.	High detection accuracies.	Failed in localization forged frames.
[23]	Multiple/double compression	Number of different coefficients between I frames of the singly and doubly compressed MPEG-2 videos.	Effective in double compression detection with same bit rate.	Performance depends on proper selection of recompression bitrate.
[24]	Region tampering	Motion residuals.	High accuracy.	Failed in forgery localization.
[25]	Region tampering	Zernike moments and 3D patch match.	Effective in forgery detecting and locating regions.	Accuracy was very low.
[26]	Region tampering	Optical flow coefficient is computed for each part.	Detected copy/move forgery effectively.	Detection failed in videos with a high amount of motion.

**Table 2:** List of Symbol abbreviations.

Symbol	Description	Symbol	Description
*T*	The input video.	*U* and *V^T^*	Unitary matrix.
*L*	Total number of all video frames.	*X_m_*	SVD feature matrix of every 3D-tensor of the selected *P_n_*.
*H × W*	Total number of rows and columns.	*Q*	Total number of 3D-tensor feature vectors of selected *Pn*.
*P_n_*	nth sub-group of a total number of N sub-groups consisting the whole *T*.	*R_m_*	Correlation between the successive 3D-tensors of the selected *P_n_*.
${\tilde{t}}_{m}$	mth 3D-tensors of a total number of M tensors consisting *P*.	*S_f_*	SVD matrix of each frame in 3D-tensor of the selected *P_n_*.
*I*	Frame matrix of each *P_n_*.	*Y_f_*	SVD feature matrix of every frame of the 3D-tensor.
*t_x_, t_y_*	Partial derivatives of the pixel intensity with coordinates (*x*,*y*) in horizontal and vertical direction.	*B*	Total number of each frame feature vectors of the selected *P_n_*.
*Corn*	Harris corner response.	*R_z_*	Correlation values between successive frames of 3D-tensors.
{(*x_c_, y_c_*)}	All Harris corner points.	*F*	Number of frames of forged 3d-tensors.

**Table 3:** Tested dataset characteristics.

NO.	Dataset Name	Length	Frame Rate	Format	Resolution
1	Akyio	300	30 fps	YUV	176 × 144
2	Hall Monitor	300	30 fps	YUV	176 × 144
3	Paris	1065	30 fps	YUV	352 × 288
4	Suzie	150	30 fps	YUV	176 × 144
5	Flower	250	30 fps	YUV	352 × 288
6	Miss America	150	30 fps	YUV	352 × 288
7	Waterfall	260	30 fps	YUV	352 × 288
8	Container	300	30 fps	YUV	352 × 288
9	Salesman	449	30 fps	YUV	176 × 144
10	Claire	494	30 fps	YUV	176 × 144
11	Bus	150	30 fps	YUV	352 × 288
12	Foreman	300	30 fps	YUV	176 × 144
13	Tempete	260	30 fps	YUV	352 × 288
14	Coastguard	300	30 fps	YUV	176 × 144
15	Carphone	382	30 fps	YUV	176 × 144
16	Mobile	300	30 fps	YUV	176 × 144
17	Mother and Daughter	300	30 fps	YUV	176 × 144
18	News	300	30 fps	YUV	176 × 144

**Table 4:** The relation between number of operations and tensor size.

	Number of Operations		
Tensor Size	F = 20 frames/tensor	F = 30 frames/tensor	F = 40 frames/tensor
F × 16 × 16	5136	7696	10,256
F × 32 × 32	20,512	30,752	40,992
F × 64 × 64	81,984	122,944	163,904
F ×100 × 100	200,100	300,100	400,100
F × 128 × 128	327,808	491,648	655,488

**Table 5:** Insertion detecting and locating performance measures of the proposed scheme for three different feature extraction methods.

	Detecting Stage									Locating Stage								
	HARRIS			GLCM			SVD			HARRIS			GLCM			SVD		
No	Precision (%)	Recall (%)	F1 Score (%)	Precision (%)	Recall (%)	F1 Score (%)	Precision (%)	Recall (%)	F1 Score (%)	Precision (%)	Recall (%)	F1 Score (%)	Precision (%)	Recall (%)	F1 Score (%)	Precision (%)	Recall (%)	F1 Score (%)
10	77	54	63	84	56	67	96	94	95	90	81	85	96	82	88	98	98	98
20	77	54	63	84	56	67	96	94	95	93	84	88	96	87	91	98	100	99
30	77	54	63	84	56	67	96	94	95	96	87	91	96	87	91	100	100	100
40	77	54	63	84	56	67	96	94	95	96	87	91	96	87	91	100	100	100
50	77	54	63	84	56	67	96	94	95	96	87	91	96	87	91	100	100	100
Avg.	77	54	63	84	56	67	96	94	95	94	85	89	96	86	90	99.2	99.6	99.4

**Table 6:** Deletion forgery detecting and locating. Results based on SVD-tensor features.

	Detecting			Locating		
No. of Forged Frames	Precision (%)	Recall (%)	F1 Score (%)	Precision (%)	Recall (%)	F1 Score (%)
<10	None	None	None	None	None	none
10	92	90	91	98	96	97
20	92	90	91	98	98	98
30	92	90	91	98	98	98
40	92	90	91	98	98	98
50	92	90	91	100	100	100
Avg.	92	90	91	98.4	98	98.2

**Table 7:** Performance comparison between proposed approach and other related methods.

Methods	Attacks Types	Precision (%)	Recall (%)	F1 Score (%)
Ref. [16]	Insertion, Deletion	89	86	87
Ref. [15]	Insertion, Deletion	95	92	93
Ref. [13]	Deletion	72	66	69
Ref. [6]	Insertion, Deletion	85	89	87
Ref. [18]	Insertion, Deletion and Duplication	98	99	98
Proposed	Insertion, Deletion	99	99	99

**Table 8:** Total time needed for Detecting and locating passive forgery.

Video	Original Length	Forgery Operation	Tampered Length	Total Time (Seconds)
1	300	10 frames inserted in 101:110	310	39.42
2	300	20 frames inserted in 50:70	320	39.49
3	250	30 frames inserted in 101:130	280	38.24
4	300	40 frames inserted in 100:140	340	39.89
5	382	50 frames inserted in 221:270	432	40.97
6	449	20 frames inserted in 201:220	469	41.40
7	300	50 frames inserted in 101:150	350	40.01
8	1065	30 frames inserted in 50:80	1086	46.24
9	300	40 frames inserted in 170:210	340	39.75
10	300	10 frames deleted in 50:59	290	27.46
11	300	20 frames deleted in 50:69	280	26.45
12	260	30 frames deleted in 160:190	230	23.89
13	449	40 frames deleted in 360:400	409	29.02
14	300	40 frames deleted in 200:240	260	25.22
15	150	10 frames deleted in 60:79	140	22.02
16	300	20 frames deleted in 100:119	280	26.44
17	250	30 frames deleted in 160:190	220	23.42
18	300	40 frames deleted in 170:210	260	25.36

## Data Availability

Not applicable.
